# Use of Non-Steroidal Anti-Inflammatory Drugs That Elevate Cardiovascular Risk: An Examination of Sales and Essential Medicines Lists in Low-, Middle-, and High-Income Countries

**DOI:** 10.1371/journal.pmed.1001388

**Published:** 2013-02-12

**Authors:** Patricia McGettigan, David Henry

**Affiliations:** 1 William Harvey Research Institute, Barts and The London School of Medicine and Dentistry, London, United Kingdom; 2 Institute for Clinical Evaluative Sciences, Toronto, Canada; 3 Department of Medicine, University of Toronto, Toronto, Canada; 4 School of Medicine and Public Health, The University of Newcastle, Newcastle, Australia; The George Institute for Global Health, Australia

## Abstract

**Background:**

Certain non-steroidal anti-inflammatory drugs (NSAIDs) (e.g., rofecoxib [Vioxx]) increase the risk of heart attack and stroke and should be avoided in patients at high risk of cardiovascular events. Rates of cardiovascular disease are high and rising in many low- and middle-income countries. We studied the extent to which evidence on cardiovascular risk with NSAIDs has translated into guidance and sales in 15 countries.

**Methods and Findings:**

Data on the relative risk (RR) of cardiovascular events with individual NSAIDs were derived from meta-analyses of randomised trials and controlled observational studies. Listing of individual NSAIDs on Essential Medicines Lists (EMLs) was obtained from the World Health Organization. NSAID sales or prescription data for 15 low-, middle-, and high-income countries were obtained from Intercontinental Medical Statistics Health (IMS Health) or national prescription pricing audit (in the case of England and Canada). Three drugs (rofecoxib, diclofenac, etoricoxib) ranked consistently highest in terms of cardiovascular risk compared with nonuse. Naproxen was associated with a low risk. Diclofenac was listed on 74 national EMLs, naproxen on just 27. Rofecoxib use was not documented in any country. Diclofenac and etoricoxib accounted for one-third of total NSAID usage across the 15 countries (median 33.2%, range 14.7–58.7%). This proportion did not vary between low- and high-income countries. Diclofenac was by far the most commonly used NSAID, with a market share close to that of the next three most popular drugs combined. Naproxen had an average market share of less than 10%.

**Conclusions:**

Listing of NSAIDs on national EMLs should take account of cardiovascular risk, with preference given to low risk drugs. Diclofenac has a risk very similar to rofecoxib, which was withdrawn from worldwide markets owing to cardiovascular toxicity. Diclofenac should be removed from EMLs.

*Please see later in the article for the Editors' Summary*

## Abstract 2

Patricia McGettigan and David Henry find that, although some non-steroidal anti-inflammatory drugs (NSAIDs) such as diclofenac are known to increase cardiovascular risk, diclofenac is included on 74 countries' essential medicine lists and was the most commonly used NSAID in the 15 countries they evaluated.

## Editors' Summary

**Background:**

Non-steroidal anti-inflammatory drugs (NSAIDs) are among the most widely used drugs. Aspirin, the first NSAID, was developed in 1897 but there are now many different NSAIDs. Some can be bought over-the-counter but others are available only with prescription. NSAIDs can help relieve short- and long-term pain, reduce inflammation (redness and swelling), and reduce high fevers. Common conditions that are treated with NSAIDs include headaches, toothache, back ache, and arthritis. NSAIDs work by stopping a class of enzymes called cyclo-oxygenases (COXs) from making prostaglandins, some of which cause pain and inflammation. Like all drugs, NSAIDs have some unwanted side effects. Because certain prostaglandins protect the stomach lining from the stomach acid that helps to digest food, NSAID use can cause indigestion and stomach ulcers (gastrointestinal complications). In addition, NSAIDs increase the risk of heart attacks and stroke to varying degrees and therefore should be avoided by people at high risk of cardiovascular diseases—conditions that affect the heart and/or blood vessels.

**Why Was This Study Done?:**

Different NSAIDs are associated with different levels of cardiovascular risk. Selective COX-2 inhibitors (e.g., rofecoxib, celecoxib, etoricoxib) generally have fewer stomach-related side effects than non-selective COX inhibitors (e.g., naproxen, ibuprofen, diclofenac). However, some NSAIDs (rofecoxib, diclofenac, etoricoxib) are more likely to cause cardiovascular events than others (e.g., naproxen). When doctors prescribe NSAIDs, they need to consider the patient's risk profile. Particularly for patients with higher risk of cardiovascular events, a doctor should either advise against NSAID use or recommend one that has a relatively low cardiovascular risk. Information on the cardiovascular risk associated with different NSAIDs has been available for several years, but have doctors changed their prescribing of NSAIDs based on the information? This question is of particular concern in low- and middle-income countries where cardiovascular disease is increasingly common. In this study, the researchers investigate the extent to which evidence on the cardiovascular risk associated with different NSAIDs has translated into guidance and sales in 15 low-, middle-, and high-income countries.

**What Did the Researchers Do and Find?:**

The researchers derived data on the relative risk of cardiovascular events associated with individual NSAIDs compared to non-use of NSAIDs from published meta-analyses of randomized trials and observational studies. They obtained information on the NSAIDs recommended in 100 countries from national Essential Medicines Lists (EMLs; essential medicines are drugs that satisfy the priority health care needs of a population). Finally, they obtained information on NSAID sales for 13 countries in the South Asian, Southeast Asian, and Asian Pacific regions and NSAID prescription data for Canada and England. Rofecoxib, diclofenac, and etoricoxib consistently increased cardiovascular risk compared with no NSAIDs. All three had a higher relative risk of cardiovascular events than naproxen in pairwise analyses. Naproxen was associated with the lowest cardiovascular risk. No national EMLs recommended rofecoxib, which was withdrawn from world markets 8 years ago because of its cardiovascular risk. Seventy-four national EMLs listed diclofenac, but only 27 EMLs listed naproxen. Diclofenac was the most commonly used NSAID, with an average market share across the 15 countries of nearly 30%. By contrast, naproxen had an average market share of less than 10%. Finally, across both high- and low-/middle-income countries, diclofenac and etoricoxib accounted for one-third of total NSAID usage.

**What Do These Findings Mean?:**

These findings show that NSAIDs with higher risk of cardiovascular events are widely used in low-/middle- as well as high-income countries. Diclofenac is the most popular NSAID, despite its higher relative risk of cardiovascular events, which is similar to that of rofecoxib. Diclofenac is also widely listed on EMLs even though information on its higher cardiovascular risk has been available since 2006. In contrast, naproxen, one of the safest in relative terms of the NSAIDs examined, was among the least popular and was listed on a minority of EMLs. Some aspects of the study's design may affect the accuracy of these findings. For example, the researchers did not look at the risk profiles of the patients actually taking NSAIDs. However, given the volume of use of high-risk NSAIDS, it is likely that these drugs are taken by many individuals at high risk of cardiovascular events. Overall, these findings have important implications for public health and, given the wide availability of safer alternatives, the researchers suggest that diclofenac should be removed from national EMLs and that its marketing authorizations should be revoked globally.

**Additional Information:**

Please access these Web sites via the online version of this summary at http://dx.doi.org/ 10.1371/journal.pmed.1001388.

- This study is further discussed in a *PLOS Medicine* Perspective by K. Srinath Reddy and Ambuj Roy
- The UK National Health Service Choices website provides detailed information on NSAIDS
- MedlinePlus provides information about aspirin, ibuprofen, naproxen, and diclofenac; it also provides links to other information about pain relievers (in English and Spanish)
- The American Heart Association has information on cardiovascular disease; Can Patients With Cardiovascular Disease Take Nonsteroidal Antiinflammatory Drugs? is a Cardiology Patient Page in the AHA journal *Circulation*
- The British Heart Foundation also provides information about cardiovascular disease and has a factsheet on NSAIDs and cardiovascular disease
- The World Health Organization has a fact sheet on essential medicines; the WHO Model List of Essential Medicines (in English and French), and national EMLs are available

## Introduction

Non-steroidal anti-inflammatory drugs (NSAIDs) are among the most widely used of therapeutic agents. Taken singly or in combination with other classes of drug, they relieve symptoms across multiple clinical indications, including short and long term pain states and a range of musculoskeletal disorders.

Serious adverse effects of NSAIDs are well understood, being related largely to their underlying mechanisms of action [1]. Extensive pharmaco-epidemiological studies and meta-analyses have documented hazards, in particular serious gastrointestinal [2],[3] and cardiovascular complications [4]–[9]. These studies have enabled some discrimination in risk between individual members of this large class of drugs, providing guidance on selection according to patient risk profiles [10]. Gastrointestinal damage can be reduced by co-prescription of proton pump inhibitors [11]. In contrast, there is no convincing evidence that low dose aspirin mitigates the cardiovascular risk of NSAIDs [12],[13]. Faced with patients at high risk of cardiovascular events, prescribers have a choice of advising against use of a NSAID or recommending a drug with a lower risk. This dilemma is not limited to doctors working in high-income countries. With the widespread use of NSAIDs and a steep rise in cardiovascular disease, it is a particular concern in low- and middle-income countries [14].

Precise summary information on cardiovascular risk with NSAIDs has been available since 2006 and current evidence suggests that there are significant differences between commonly used members of the class [4]–[9],[13]. There are strong reasons for choosing low risk NSAIDs in those at high risk of cardiovascular events. Our interest here is the extent to which this is reflected in Essential Medicines Lists (EMLs) and in data on sales across several countries.

## Methods

### Estimating Cardiovascular Risk with Individual Drugs

We ranked NSAIDs by cardiovascular risk (with non-use as reference) using relative risk (RR) values derived from published meta-analyses of randomised trials and controlled observational studies that reported RR for three or more individual drugs [4]–[9]. Data on pairwise comparisons of individual agents, representing the least confounded comparisons of RR, were obtained from the most recent meta-analysis [9].

### Essential Medicines Lists

Essential medicines are those satisfying the priority health care needs of the population. They are selected with due regard to public health relevance, evidence on efficacy and safety, and comparative cost-effectiveness. We determined the NSAIDs recommended by the World Health Organization (WHO) in its Model List of Essential Medicines [15], and by individual countries with published national EMLs [16]. National EMLs are informed by the WHO Model List of Essential Medicines and modified to reflect national health care priorities [17]. For the countries with published EMLs, we compared the information on cardiovascular risk with individual NSAIDs and their inclusion in the country EML.

### Measuring Sales of NSAIDs in Different Countries

Intercontinental Medical Statistics Health (IMS Health) tracks over 80% of global pharmaceutical use by sampling individual country sales through multiple supply routes to retail pharmacies and hospitals (http://www.imshealth.com/portal/site/ims/). These sales include both indirect sales from wholesalers and direct sales from manufacturers. In some countries, hospital audits are based on data sourced from hospital pharmacies. In each country, the sampling data are projected to estimate sales for the whole country. We purchased data from IMS Health on the mass of individual NSAIDs sold in 2011 in 13 countries in the South Asian, Southeast Asian, and Asian Pacific regions (Multinational Integrated Data Analysis, MIDAS). The countries included in the analyses were: Australia, Bangladesh, China, China (Hong Kong), Indonesia, Malaysia, New Zealand, Pakistan, Philippines, Singapore, Taiwan, Thailand, and Vietnam. The data reflected retail pharmacy and hospital sales in all countries except Bangladesh and Pakistan (retail pharmacy sales) and China (hospital sales). Defined daily doses (DDD), established by the WHO Collaborating Centre for Drug Statistics Methodology (WHOCC), permit comparisons of use between different drugs and across different countries [18]. We calculated the numbers of DDD of individual NSAIDs for each country using the values published by the WHOCC [18].

Data on NSAID prescriptions dispensed in the community in England during 2011 were obtained from public prescription cost analysis reports [19]. For Canada, we purchased data on NSAID prescriptions dispensed in the community during 2011 from IMS Brogan (IMS Brogan Inc., Ottawa, Canada). We did not have sufficient information to convert prescription data to DDD, but assumed that the proportionality of market shares for individual NSAIDs calculated from prescriptions would be equivalent to that derived from sales data.

## Results

### Cardiovascular Risk with Individual NSAIDs

The meta-analyses were fairly constant in their findings (Table 1). The NSAIDs that had consistently higher cardiovascular risks (RRs) were rofecoxib, etoricoxib, and diclofenac. All were found to have a higher RR than naproxen in pairwise analyses in the most recent published meta-analysis [9]. Indometacin and meloxicam had moderately elevated RR values that were significantly greater than naproxen [9]. Etodolac was found to have an elevated risk but in pairwise analysis, it did not have a statistically significantly higher RR than naproxen [9]. Celecoxib and ibuprofen were associated with elevated RR values when used in clinical trials in high doses but not in the lower doses typically used in the community. We judged naproxen to have the lowest risk. Five of the six meta-analyses found it to be risk-neutral (Table 1). We classified rofecoxib, etoricoxib, and diclofenac as “high risk” drugs for the purpose of analysis. This is conservative in that other NSAIDs could also be considered high risk.

**Table 1:** Summary of relative risk estimates for cardiovascular events with individual NSAIDs (versus non-use).

NSAID	Serious Cardiovascular Events; RR (95% CI) Versus Non-use of NSAIDs					
	Observational Studies (Outcomes)				Randomised Studies (Outcomes)	
	Hernandez-Diaz et al., 2006 [4] (AMI)	Singh et al., 2006 [5] (AMI)	McGettigan and Henry, 2006 [6] (CV Events)	McGettigan and Henry, 2011 [9] (CV Events)	Trelle et al., 2011 [7] (APTC Composite Outcomes)	Kearney et al., 2006 [8] (CV Events)
**Etoricoxib**	nr	nr	nr	2.05 (1.45–2.88)	1.53 (0.74–3.17)	nr
**Etodolac**	nr	nr	nr	1.55 (1.28–1.87)	nr	nr
**Rofecoxib**	1.27 (1.12–1.44)	nr	1.35 (1.15–1.59)	1.45 (1.33–1.59)	1.44 (1.00–1.99)	1.42 (1.13–1.78) (with celecoxib)^a^
**Diclofenac**	1.39 (1.18–1.64)	1.38 (1.22–1.57)	1.40 (1.16–1.70)	1.40 (1.27–1.55)	1.60 (0.85–2.99)	1.63 (1.12–2.37)
**Indometacin**	nr	nr	1.30 (1.07–1.60)	1.30 (1.19–1.41)	nr	nr
**Meloxicam**	nr	nr	1.25 (1.00–1.55)	1.20 (1.07–1.33)	nr	nr
**Ibuprofen**	1.01 (0.89–1.15)	1.11 (1.06–1.17)	1.07 (0.97–1.18)	1.18 (1.11–1.25)	2.26 (1.11–4.89)	1.51 (0.96–2.37)
**Celecoxib**	0.97 (0.86–1.08)	nr	1.06 (0.91–1.23)	1.17 (1.08–1.27)	1.43 (0.94–2.16)	1.42 (1.13–1.78)(with rofecoxib)^a^
**Naproxen**	0.98 (0.87–1.11)	0.99 (0.88–1.11)	0.97 (0.87–1.07)	1.09 (1.02–1.16)	1.22 (0.78–1.93)	0.92 (0.67–1.26)
**Piroxicam**	nr	nr	1.06 (0.70–1.59)	1.08 (0.91–1.30)	nr	nr

a celecoxib and rofecoxib analysed together.

AMI, acute myocardial infarction; APTC, Anti-Platelet Trialists Collaboration; CV, cardiovascular; nr, not reported.

### EML Inclusions

The WHO Model List of Essential Medicines includes three drugs, paracetamol, acetyl salicylic acid (aspirin), and ibuprofen, in the category “non-opioids and non-steroidal anti-inflammatory medicines.” Of 100 countries with EMLs published on the WHO website, most included fewer than six agents in this class. The NSAIDs most commonly recommended were: aspirin (88 countries), ibuprofen (90 countries), diclofenac (74 countries), indometacin (56 countries), and naproxen (27 countries) (Table S2). Significantly, 51 of the countries that listed diclofenac did not list naproxen. Selective cyclooxygenase-2 (cox-2) inhibitors were included on the EMLs of 12 countries. Of 86 EMLs published or updated since 2007, diclofenac was listed on 74, naproxen on 27.

### Patterns of NSAID Use

**Figure 1:**
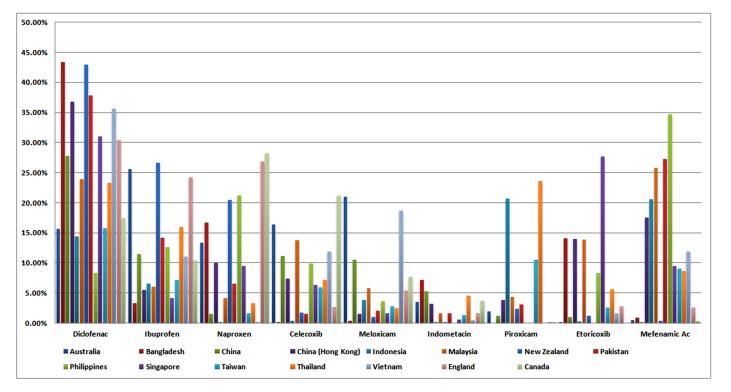
Individual NSAID defined daily doses (DDD) expressed as a percentage of total NSAID DDD sales in each country in 2011. Data reflect retail pharmacy and hospital sales in all countries except Bangladesh and Pakistan [retail pharmacy sales], China [hospital sales], and England and Canada [prescription sales only].

Figure 1 presents the market shares of the nine most widely sold NSAIDs in 15 countries. The analyses are presented in Table 2. Full data are provided in Table S1. Diclofenac was the most popular NSAID, with a market share almost equal to that of the next three most popular NSAIDs combined (ibuprofen, mefenamic acid, naproxen). There was no documented use of rofecoxib. Etoricoxib was commonly sold in Bangladesh, Malaysia, Hong Kong, and Singapore. “High risk” NSAIDs (diclofenac and exoricoxib) comprised about one-third of the market across the 15 countries (median 33.2%, range 14.7–58.7%), and this proportion did not differ between high- and low-income states (Table 2).

**Table 2:** Analysis of use of selected NSAIDs in 15 countries.

NSAID	Individual NSAID Use Expressed as Percent of Total NSAID Sales in All Countries in 2011		
	Median	Maximum	Minimum
**Diclofenac**	27.80%	43.40%	8.30%
**Ibuprofen**	11.00%	26.60%	3.30%
**Naproxen**	9.40%	28.20%	0.00%
**Mefenamic Acid**	9.10%	34.70%	0.00%
**Celecoxib**	7.20%	21.20%	0.20%
**Meloxicam**	3.60%	21.00%	0.30%
**Piroxicam**	3.10%	23.60%	0.00%
**Etoricoxib**	2.80%	27.60%	0.20%
**Indometacin**	3.67%	7.20%	0.00%
**Ketoprofen**	1.10%	9.50%	0.20%
**High Risk NSAIDs** a	33.20%	58.69%	14.65%
HMIC	31.10%	58.70%	15.80%
LMIC	37.30%	57.50%	14.70%

Percentage refers to proportion of total NSAID sales in all countries studied. HMIC (high-/high middle-income countries): Australia, China, China (Hong Kong), Malaysia, New Zealand, Singapore, Taiwan, Thailand, UK/England, Canada; LMIC (low-/low middle-income countries): Bangladesh, Indonesia, Pakistan, Philippines, Vietnam.

a Diclofenac, etoricoxib.

## Discussion

NSAIDs with a high risk of cardiovascular complications are widely used. Diclofenac and etoricoxib together account for approximately one-third of all sales of NSAIDs in the 15 countries included in our analysis. There was no difference between high- and low-income countries. Diclofenac was by far the most popular NSAID, despite having an RR identical to rofecoxib [9], which was withdrawn from world markets 8 years ago owing to cardiovascular toxicity [20]. The information on cardiovascular risk associated with diclofenac has been available to regulators, writers of guidelines and essential medicines lists, and prescribers for at least 5 years [4]–[9]. Calls have been made for its withdrawal [21]. High levels of sales as recently as 2011 suggest that none of this information has resulted in effective action. There has been a slow decline in prescription numbers in England, Australia, and Canada since 2006 (Figure S1), but it remains popular in all three countries, particularly in England where it is the single most-prescribed NSAID (Table S1). While the popularity of diclofenac in high-income countries is well known, to our knowledge this is the first report that highlights the risks associated with its dominant market position in low- and middle-income countries.

Etoricoxib is the other high risk NSAID that features in this study. While there is limited information on its cardiovascular risk, an updated meta-analysis published by us in 2011 found a doubling of cardiovascular risk compared with non-use [9]. It was significantly more harmful than ibuprofen and naproxen in pairwise comparisons. In a large head-to-head randomised clinical trial, it had an identical cardiovascular risk to diclofenac [22]. In the current study, etoricoxib accounted for 28% of NSAID sales in Singapore, and 14% in Bangladesh, Hong Kong, and Malaysia. In England, it is prescribed as often as celecoxib (Table S1), but it is not licensed in North America.

Based on meta-analyses of randomised and non-randomised studies, the greatest amount of evidence supports naproxen as the safest choice to minimize cardiovascular risk. However, it was listed in only 27 out of 86 national EMLs published or updated since 2007. In contrast, diclofenac was included on 74 of these EMLs. On average, diclofenac was used three times as frequently as naproxen. In other words, evidence on the relative cardiovascular safety of this drug has failed to translate into appropriate selection for EMLs or usage. The WHO Model List of Essential Medicines provides limited guidance for selection of NSAIDs on EMLs [15]. It includes aspirin and ibuprofen, but offers no advice on their safety or cost-effectiveness relative to each other or to other NSAIDs.

There are a number of limitations to this work. Most obviously, we do not have information on the risk profiles of patients taking NSAIDs. However, the large and consistent volumes of use of high risk NSAIDs make it very likely that these drugs are being taken by substantial numbers of individuals at high risk of serious cardiovascular events. We relied on sales data for 13 countries and prescription sales for England and Canada. Sales data provide the most comprehensive estimates capturing non-prescription and hospital use in addition to community prescribing, although coverage of all sectors was variable in our study. We could not analyse prevalence of use or dosage, and while it is possible that duration of treatment varies between individual drugs, we don't think this is likely to distort greatly the patterns we have observed in the overall sales data. Importantly, the increase in cardiovascular risk has been reported very early in the course of diclofenac treatment [9],[21].

The findings here have significant implications for public health. For instance, in China the age- and sex-standardised death rate from cardiovascular disease is estimated to be 312/100,000 for males and 260/100,000 for females [23]. Diclofenac is the most commonly used NSAID in hospitals in China. We assume community use follows a similar pattern. If it were taken by only 1% of China's population of approximately 1.3 billion annually, based on the relative risk calculations from meta-analyses it could cause 14,000 additional unintended deaths. These deaths are preventable—lower risk NSAIDs, including naproxen and low-dose ibuprofen, are widely available and are equally efficacious [24],[25]. Both are available as generic products.

There is increasing regulatory concern about diclofenac. The European Medicines Agency has just commenced (as of October 2012) a new review of its cardiovascular safety [26]. In low- and middle-income countries, national EMLs are authoritative influences on drug choice, being used as the basis for procurement of safe, cost-effective medicines for public reimbursement and to guide local medicines production [17]. NSAID recommendations on national EMLs should be based on the optimum balance of benefit and harm and give preference to low risk drugs, in particular to ibuprofen and naproxen. Diclofenac has no advantage in terms of gastrointestinal safety [11] and it has a clear cardiovascular disadvantage [9]. Given the availability of safer alternatives, diclofenac should be de-listed from national EMLs. There are strong arguments to revoke its marketing authorisations globally.

## Supporting Information

Figure S1 **NSAID prescriptions dispensed in the community in England, Australia, and Canada.** Data sources: England, National Health Service Prescription Cost Analysis Reports; publicly available up to and including 2011 (http://www.ic.nhs.uk/pubs/prescostanalysis2011 Accessed 12November 2012); Australia, Australian Statistics on Medicines Reports, publicly available up to and including 2009 (http://www.health.gov.au/internet/main/publishing.nsf/Content/health-pbs-general-pubs-asm.htm) (accessed 12 November 2012); Canada, data estimates of prescription numbers dispensed annually, 2007–2011 inclusive, in the community in Canada, purchased from Intercontinental Medical Statistics (IMS), IMS Brogan, a unit of IMS Health, Toronto, Canada (http://www.imshealth.com/portal/site/ims?CURRENT_LOCALE=en_ca) (accessed 12 November 2012). **Cox-2-selective**, all “coxib” NSAIDs available each year in each country including celecoxib, etoricoxib, lumiracoxib, rofecoxib, valdecoxib; **non-selective NSAIDs, a**ll NSAIDs except Cox-2 selective (coxibs) and meloxicam.
(TIF)

Table S1 **Use of individual NSAIDs expressed as a percentage of total NSAID use in each country in 2011. Use is expressed as sales of defined daily doses (DDD, in millions) for all countries except England and Canada where it is expressed as millions of prescriptions dispensed in the community.**
(DOCX)

Table S2 **NSAIDs listed on national Essential Medicines Lists; World Health Organization (2012) Essential Medicines Selection.**
(XLSX)

## Abbreviations

EML: Essential Medicines List
NSAID: non-steroidal anti-inflammatory drug
RR: relative risk
WHO: World Health Organization
