# Supplementary figures and images for: Use of Non-Steroidal Anti-Inflammatory Drugs That Elevate Cardiovascular Risk: An Examination of Sales and Essential Medicines Lists in Low-, Middle-, and High-Income Countries
Source: PLoS Med. 2013 Feb 12;10(2):e1001388. doi: 10.1371/journal.pmed.1001388 (PMC3570554; doi:10.1371/journal.pmed.1001388)

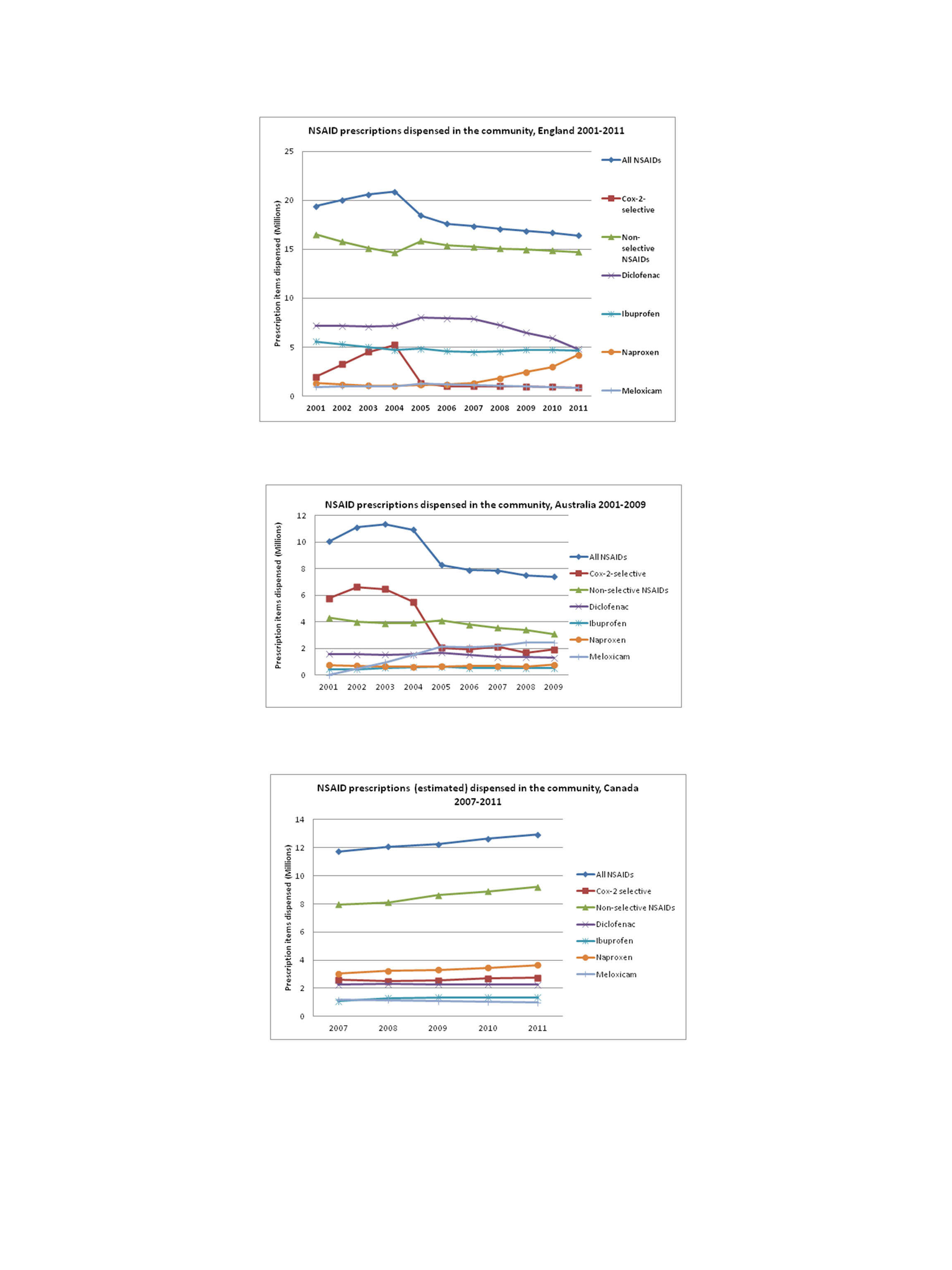

Supplement: Figure S1 — NSAID prescriptions dispensed in the community in England, Australia, and Canada. Data sources: England, National Health Service Prescription Cost Analysis Reports; publicly available up to and including 2011 (http://www.ic.nhs.uk/pubs/prescostanalysis2011 Accessed 12November 2012); Australia, Australian Statistics on Medicines Reports, publicly available up to and including 2009 (http://www.health.gov.au/internet/main/publishing.nsf/Content/health-pbs-general-pubs-asm.htm) (accessed 12 November 2012); Canada, data estimates of prescription numbers dispensed annually, 2007–2011 inclusive, in the community in Canada, purchased from Intercontinental Medical Statistics (IMS), IMS Brogan, a unit of IMS Health, Toronto, Canada (http://www.imshealth.com/portal/site/ims?CURRENT_LOCALE=en_ca) (accessed 12 November 2012). Cox-2-selective, all “coxib” NSAIDs available each year in each country including celecoxib, etoricoxib, lumiracoxib, rofecoxib, valdecoxib; non-selective NSAIDs, all NSAIDs except Cox-2 selective (coxibs) and meloxicam. (TIF) [file pmed.1001388.s001.tif]
