# Supplementary material for: Use of Non-Steroidal Anti-Inflammatory Drugs That Elevate Cardiovascular Risk: An Examination of Sales and Essential Medicines Lists in Low-, Middle-, and High-Income Countries
Source: PLoS Med. 2013 Feb 12;10(2):e1001388. doi: 10.1371/journal.pmed.1001388 (PMC3570554; doi:10.1371/journal.pmed.1001388)
Supplement: Table S1 — Use of individual NSAIDs expressed as a percentage of total NSAID use in each country in 2011. Use is expressed as sales of defined daily doses (DDD, in millions) for all countries except England and Canada where it is expressed as millions of prescriptions dispensed in the community. (DOCX) [file pmed.1001388.s002.docx]

| **SUPPORTING INFORMATION**  **TABLE** | **Low/ low middle income countries** | | | | | | **High / high middle income countries** | | | | | | | | | |
| --- | --- | --- | --- | --- | --- | --- | --- | --- | --- | --- | --- | --- | --- | --- | --- | --- |
|  | **Bangladesh** | **Indonesia** | **Pakistan** | **Philippines** | **Vietnam** | | **Australia** | **China** | **China (Hong Kong)** | **Malaysia** | **New Zealand** | **Singapore** | **Thailand** | **Taiwan** | **England*** | **Canada*** |
| **NSAID usage in DDDs (Millions) 2011** | **543.7** | **1173.1** | **1309.1** | **199.3** | **103.8** | | **297.4** | **346.1** | **63.9** | **111.5** | **63.5** | **31.3** | **447.2** | **525.8** | **15.6*** | **12.9*** |
| **Diclofenac** | 43.4% | 14.4% | 37.9% | 8.3% | 35.7% | | 15.6% | 27.8% | 36.8% | 24.0% | 43.0% | 31.0% | 23.3% | 15.7% | 30.4% | 17.4% |
| **Ibuprofen** | 3.3% | 6.5% | 14.1% | 12.6% | 11.0% | | 25.6% | 11.5% | 5.5% | 6.0% | 26.6% | 4.2% | 16.0% | 7.2% | 24.3% | 10.4% |
| **Naproxen** | 16.7% | 0.0% | 6.6% | 21.2% | 0.1% | | 13.3% | 1.5% | 10.0% | 4.2% | 20.5% | 9.4% | 3.3% | 1.6% | 26.8% | 28.2% |
| **Celecoxib** | 0.2% | 0.3% | 1.5% | 9.9% | 11.9% | | 16.4% | 11.1% | 7.4% | 13.8% | 1.7% | 6.4% | 7.2% | 5.9% | 2.7% | 21.2% |
| **Meloxicam** | 0.3% | 3.8% | 2.0% | 3.6% | 18.7% | | 21.0% | 10.6% | 1.5% | 5.8% | 1.0% | 1.6% | 2.5% | 2.7% | 5.4% | 7.7% |
| **Indometacin** | 7.2% | 0.0% | 1.6% |  | 0.4% | | 3.5% | 5.2% | 3.2% | 1.7% | 0.1% | 0.6% | 4.6% | 1.3% | 1.6% | 3.7% |
| **Piroxicam** |  | 20.7% | 3.1% |  |  | | 2.0% | 1.1% | 3.8% | 4.4% | 2.3% |  | 23.6% | 10.5% | 0.0% | 0.1% |
| **Etoricoxib** | 14.1% | 0.3% |  | 8.3% | 1.6% | | 0.2% | 0.9% | 13.9% | 13.9% | 1.2% | 27.6% | 5.6% | 2.5% | 2.8% |  |
| **Mefenamic Acid** | 0.9% | 20.5% | 27.3% | 34.7% | 11.9% | | 0.5% | 0.0% | 17.6% | 25.8% | 0.3% | 9.5% | 8.6% | 9.1% | 2.6% | 0.4% |
| **Phenylbutazone** |  | 30.4% |  |  |  | |  |  |  |  |  |  | 2.4% |  |  |  |
| **Ketoprofen** | 1.4% | 0.7% |  |  | 1.1% | | 1.7% | 1.4% | 2.0% |  | 0.4% | 9.5% |  | 0.7% | 0.4% | 0.2% |
| **Nimesulide** |  |  |  |  |  | |  | 13.6% |  |  |  |  |  |  |  |  |
| **Loxoprofen** |  |  |  |  |  | |  | 11.9% |  |  |  |  |  |  |  |  |
| **Flurbiprofen** |  |  | 5.2% |  |  | |  |  |  |  |  |  |  | 38.0% | 0.1% | 0.2% |
| **Ketorolac** | 6.8% | 0.8% |  |  | 0.0% | |  | 1.0% | 0.1% |  | 0.0% |  |  | 0.8% |  | 3.9% |
| **Tenoxicam** | 3.3% |  |  |  | 0.4% | |  |  |  |  | 2.7% |  |  | 0.1% |  | 0.0% |
| **Etodolac** |  |  |  |  | 0.7% | |  |  | 0.1% |  |  |  |  | 1.4% | 1.4% | 0.1% |
| **Proportion of total NSAID use comprised by 'high' risk NSAIDs: etoricoxib, diclofenac** | | | | | | | | |  |  |  |  |  |  |  |  |
|  | **57.5%** | **14.7%** | **37.9%** | **16.6%** | | **37.3%** | **15.8%** | **28.7%** | **50.7%** | **37.8%** | **44.2%** | **58.7%** | **28.9%** | **18.3%** | **33.2%** | **17.4%** |
|  | | | | | | | | | | |  |  |  |  |  |  |

**Supporting Information Table. Use of individual NSAIDs expressed as a percentage of total NSAID use in each country in 2011. Use is expressed as sales of defined daily doses (DDDs, in millions) for all countries except England* and Canada* where it is expressed as millions of prescriptions dispensed in the community.**
